# Preshooting Electroencephalographic Activity of Professional Shooters in a Competitive State

**DOI:** 10.1155/2021/6639865

**Published:** 2021-01-31

**Authors:** Jie Zhang, Yunxu Shi, Chienkai Wang, Chunmei Cao, Changshui Zhang, Linhong Ji, Jia Cheng, Fangfang Wu

**Affiliations:** ^1^State Key Laboratory of Tribology, Department of Mechanical Engineering, Tsinghua University, Beijing, China; ^2^School of Electromechanical and Automotive Engineering, YanTai University, Yantai, Shandong, China; ^3^Division of Sports Science and Physical Education, Tsinghua University, Beijing, China; ^4^Department of Automation, Tsinghua University, Beijing, China

## Abstract

This study investigated the influence of competitive state on cerebral cortex activity of professional shooters with 10 m air rifle before shooting. Generally, professional athletes have higher neural efficiency compared with ordinary people. We recruited 11 national shooters to complete 60 shots under both noncompetitive and competitive shooting conditions, and simultaneously collected their electroencephalogram (EEG) and electrocardiogram (ECG) information. Theta, alpha, and beta power were computed in the last three seconds preceding each shot from average-reference 29-channel EEG, while EEG characteristics under two conditions were analyzed. The results showed a significant linear correlation between shooting accuracy and EEG power of anterior frontal, central, temporal, and occipital regions in beta and theta bands. In addition, the theta power in occipital regions, alpha power in frontal-central and left occipital regions, and beta power in frontal and mid-occipital regions were higher than those in noncompetitive state. However, heart rate (HR) and shooting accuracy did not change significantly under the two conditions. These findings reveal the changes of cortical activity underlying competition shooting as well as providing further understanding of the neural mechanisms of the shooting process and lay a foundation for the subsequent neuromodulation research.

## 1. Introduction

It is well known that athletes may suffer different pressures during competition, which may affect their sports performance. Shooting is a very popular sport, and the relationship between shooting performance and neural activity is a classic topic in the field of kinematics. The shooting performance of the subjects can be evaluated and improved from a neurological perspective. A lot of literature has referred to energy consumed by neural activity as neural efficiency [1, 2] and some basic research has shown that the efficiency of cortical processing can be used to describe superior motor performance. For example, when comparing the differences in neural activity between expert athletes and novice athletes, it is found that the heart rate (HR) and electroencephalographic (EEG) power of expert athletes decrease greatly in the last few seconds before the exercise. It showed that expert athletes can achieve better results with less neural activity [3–7]. Based on the tenets of the multi-action plan (MAP) model, some research explored the neural marker underlying optimal and suboptimal performance experiences of shooters, and suggested that the performance of each functional movement is a combination of different cognitive processes and unique neural patterns, and the optimal and suboptimal performance states were related to different cortical patterns. Different performance states are associated with unique neural patterns, so that expert shooters can realize automatic processing and controlled state switching of the brain according to different needs [8–11]. What's more, Gong et al. analyzed the correlations between EEG characteristics and the shooting performance by various methods (band power, eyes open-close ERD/ERS, coherence, and brain network topology), and identified multiple correlations between resting-state EEG characteristics and shooting performance [12]. In 2018, Gong et al. found a significant negative correlation between shooting performance and functional coupling between the prefrontal, frontal, and temporal regions of the right brain in the Beta1 and Beta2 frequency bands [13]. These studies provide theoretical support for us to further explore the relationship between shooting performance and brain activity. On the other hand, the research on improving sports performance by neural regulation has been applied in the field of sports science. For instance, transcranial direct current stimulation (tDCS), a noninvasive technique, can change the excitability of neurons by stimulating specific locations of the brain, which in turn regulates brain function [14].

In the field of sports science, EEG has been widely used to evaluate athletic ability [14–19], and the analysis of some specific frequency bands makes use of various psychological processes. Some studies have reported that higher frontal theta activity is related to excellent performance in goal-directed tasks [7, 17, 20]. Compared with novices, expert shooters have higher theta activity located at the anterior cingulate area and medial frontal cortex. It showed that experts can correctly focus attention on the moment of the trigger pull. This provides strong evidence for Cavanagh's research, which suggested that the theta band activity over the mid-frontal cortex may be used to communicate the need for cognitive control and subsequently implemented to control across disparate brain regions [21]. On the other hand, the study of EEG activity suggests that there was an inverse relationship between alpha power and cortical activity [3]. The traditional explanation was that a decrease of alpha power means an increase of cortical activation in specific tasks [3, 22, 23]. Many studies have shown that higher occipital alpha power and lower central alpha power were characteristics of professional skills and successful performance. For instance, in 2001, Loze, Collins, and Holmes found that occipital alpha power increased significantly before best shots, whereas it decreased before triggering during worst shots [4, 24, 25]. These findings suggest that the theta and alpha power of specific brain regions are related to the accuracy and professionalism of shooting. Lu et al. found that a larger oscillatory activity in both the mid-frontal theta and parieto-occipital alpha in experts suggested an adaptive neural preparatory process with highly developed motor skills in complex tasks, characterized by high attentional demands, anticipation of uncertainty, and the integration of multiple visual cues [26]. This provides a theoretical support for us to study the neural activities in the process of shooting preparation for competitive tasks.

Some researchers have proposed that competition changes the environment for athletes [27], and from the point of view of improving motor performance, the neural basis underlying competition has been studied by using different methods. Among them, in 2014, Antonis et al. proposed a self-talk intervention for competitive sport performance and reported that the performance of the group participating in self-talk is significantly improved compared to the control group, which provides a new direction for the study of improving motor performance in a competitive environment [15]. Moreover, in 2004, Jean Decety et al. suggested a work on a functional magnetic resonance imaging (fMRI) investigation, where they studied human cooperation and competition from the perspective of neuroimaging. Participants completed experimental tasks by playing online games, and the results showed that compared with independent games, cooperation and competition were related to a common set of neural regions. Cooperation was associated with right orbitofrontal involvement, while competition was associated with increased prefrontal activity [28]. In a shooting competition, the cerebral-cortical activity associated with winning and losing were studied. The psychomotor processes underlying winning and losing were investigated by examining spectral power and coherence estimates derived from the EEG data; as a result, the winner displayed a global decrease in high alpha power, and the self-reported confidence of the winners was greater than that of the losers [29]. However, there is no paper on the effect of competition environment on the EEG of professional shooters with rich experience. Competition can result in better sports performance. To throw some light on the neurophysiological adaptations related to competition, we examined EEG activity during competitive shooting. We hoped to find the brain region associated with specific shooting performance and lay the foundation for further research on the improvement of shooting skill.

In this study, in order to explore the physiological impact of competition on shooters, we selected excellent air rifle shooters to complete personal shooting and “one-on-one” competitive shooting tasks in a real 10-meter air rifle shooting hall. Participants needed to complete 60 effective shots in each experiment, while recording their EEG and ECG signals. The aims of the work were twofold. The first aim was to examine the changes of neurophysiological activity during competitive shooting. We hypothesized that competition would change the activation of the brain regions in theta, alpha, and beta bands. The second aim was to explore the relation between EEG activity and shooting performance. We speculated that there was a correlation between the distribution of EEG power and the shooting score.

## 2. Experimental Design

### 2.1. Subjects

Eleven college students who were national (5 males, 6 females) 10-meter air rifle shooters were recruited in this study. They were aged between 18 and 27 years (Mean=22, SD=2.82), and had a training and competition experience of 8–15 years. They abstained from alcohol and other drinks that may impact shooting performance for 24 h before the experiment. All subjects were right-handed and their eye sight was normal. The subjects understood the experimental process and gave their informed consent to participate in this research before the experiment. This study has been approved by the ethics committee of Tsinghua University.

### 2.2. Shooting Task

The experiments were conducted at a shooting training hall in Tsinhua university, and the experiments were conducted after the national championship. Subjects completed a shooting task in a competitive experiment and noncompetitive conditions, which were completed in different periods of time. The competitive experiment was designed as a match according to the international competition standard, and a system with reward and punishment was set up. One subject competes with another subject (competition condition) and the winner gets a reward of 100 yuan. In order to ensure the preciseness of the experiment, the match list was made by the coach according to the usual training results of the athletes. During the experiment, the coach was invited to watch and report the score of each shot to two subjects. While the noncompetitive experiment was an individual shooting task, subjects were alone in the shooting rang (training condition). The subjects did the noncompetitive experiment, followed by six competition groups. Each subject performed 60 shots in 75 minutes at their own pace. Subjects used their own rifle, aiming at the outside of a target 10 m away. All of them were in the standing position and used their right hand shooting stance. Each participant was instructed to perform a normal shooting skill, and the duration of the experiment was 2-2.5 hours for every participant. EEG and ECG signals were recorded during the experiment, simultaneously.

### 2.3. Experimental Procedures

**Figure 1:**
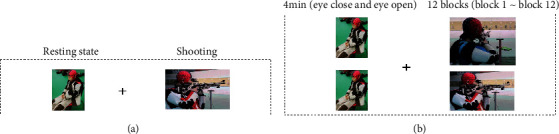
The experimental process. For each block, the subjects fired 5 shots. (a) noncompetition. (b) competition.

Before the experiment began, the experimenters required were as follows: (1) testers introduced the purpose of the study; (2) testers introduced the experimental equipment and operation process; and (3) subjects signed informed consent and filled in the basic personal information form. Following instrumentation for physiological recording, subjects were asked to sit comfortably and resting-state EEG recordings (eyes closed and eyes open) were conducted for about 4 min. Then, subjects calibrated their rifles and practiced shooting for about 10 min. Finally, subjects began the shooting tests and a set of five consecutive shots were fired within each of the 12 blocks, 60 shots in total (there was a 1 min break between blocks). The experimental flow is shown in Figure 1.

### 2.4. Physiological Signal Acquisition

**Figure 2:**
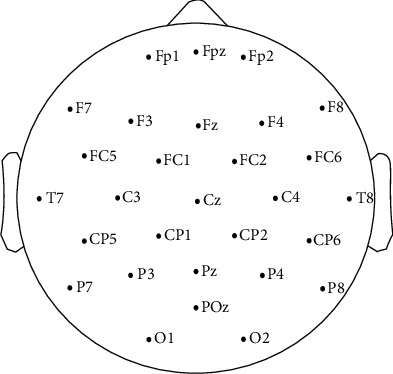
The placement of electrodes using the standard 10–20 system.

The EEG was recorded with way of Eego^TM^mylab system produced by ANT company, and a Holter-32D EEG amplifier was used as well. Placement of the recording electrodes was in accordance with the international 10–20 system Figure 2. The electrode positions were Fp1, Fpz, Fp2, F7, F3, Fz, F4, F8, FC5, FC1, FC2, FC6, M1, T7, C3, Cz, C4, T8, M2, CP5, CP1, CP2, CP6, P7, P3, Pz, P4, P8, POz, O1, and O2, with a ground electrode placed at the AFz and reference electrode at Cpz. During the experiments, the impedance of all electrodes was kept below 5 *k*Ω, and the sampling frequency was 1000 Hz. The EEG signals were processed using the EEGLAB toolbox and MATLAB, and re-referenced to the average of all channels. To eliminate noise interference, EEG signals were 0.5–45 Hz pass band filtering [30]. After filtering, the EEG data of 60 shots were divided into 12 blocks for Independent Component Analysis (ICA). In order to reduce the impact of blinking on EEG signals, eye artifact correction was applied [29]. Artificial visual inspection was carried out on each segment of data, in which eye movements and other recognizable components of nonneural activity were removed. To obtain more stable and reliable EEG characteristic parameters, the superimposition average method was adopted in calculation. In one experiment, after each subject completed 60 shots, 60 EEG data segments were extracted. The portable wearable dynamic ECG recording and analysis system was used for ECG signal acquisition, which was placed two centimeters under the left clavicle at an angle of 45 degrees to the left and down (armpit) and collected synchronously during the shooting. The ECG signals were processed using the ECGLabPro to detect the change of HR in the shooting process.

## 3. Data Processing and Analysis

### 3.1. HR (Heart Rate)

HR at the moment of shooting was derived from the heart beat interval corresponding to each shot. Ratings were divided into noncompetitive condition (1–12 blocks) and competitive condition (13–24 blocks) before shooting. Values were averaged across each block to evaluate the change of HR during shooting.

### 3.2. Shooting Accuracy

Shooting accuracy was evaluated using the shooting score, and the score of each shot was recorded through a computer target-scoring system (scat optical shooting training system of Russia). Target rings were scored ranging from 6 (outer ring) to 10.9 (center of the target).

### 3.3. EEG Power

The EEG data format after preprocessing was analyzed within the MATLAB 2015a software. The power was computed in the 3 seconds preceding each shot from average-reference 29-channel EEG and averaged across time to generate values within 3 epochs: −3000 to −2000 ms, −2000 to −1000 ms, and −1000 to 0 ms. Alpha power in the closed eye-resting-state EEG data were computed to define each subject's Individual Alpha Frequency (IAF) [31]. The frequency ranges were redefined according to IAF: theta frequency range as IAF − 6 Hz to IAF − 4 Hz, alpha as IAF − 2 Hz to IAF+2 Hz, and beta as IAF+3 Hz to IAF+10 Hz. The FFT method with a Hanning window was used to calculate the power spectrum across the 3 defined frequency ranges for all 29 electrode channels (removal of binaural mastoid). The power was averaged across selected channels to yield values for each region. Taking into consideration the influence of inter-individual differences, all values were subjected to a median-scaled log transformation [16, 32].

### 3.4. Statistical Analysis

Multivariate analysis of variance (MANOVA) for repeated measures was conducted to evaluate the hypothesis of this study. SPSS25.0 software was used for statistical analysis, and the confidence level was set at 0.95.

#### 3.4.1. The Effect of Competition on EEG Power

HR and shooting accuracy were subjected to paired-sample t-tests (competition versus noncompetition). To ensure frequency specificity, theta power, alpha power, and beta power were subjected to repeated-measures MANOVA with the factors Condition, Epoch, and Region. Theta power was subjected to separate 2 × 3 × 5(Condition × Epoch × Region) ANOVAs. Condition (noncompetition and competition), Epoch (−3000 to −2000 ms, −2000 to −1000 ms, −1000 to 0 ms), and Region (prefrontal: Fp1, Fpz, Fp2; frontal-central: FC1, FC2; left temporal: T7, P7; right temporal: T8, P8; occipital: POz, O1, O2) were the within-subjects factors. Alpha power was subjected to separate 2 × 3 × 4(Condition × Epoch × Region) ANOVAs. Condition (noncompetition and competition), epoch (−3000 to −2000 ms, −2000 to −1000 ms, and −1000 to 0 ms), and region (frontal-central: FC1, FC2; left temporal: T7, P7; left occipital: O1; and right occipital: O2) were the within-subject factors. Beta power was subjected to separate 2 × 3 × 4(Condition × Epoch × Region) ANOVAS, and also only for Region (prefrontal: Fp1, Fpz, Fp2; mid-frontal: F3, Fz, F4; frontal-central: FC1, FC2; and mid-occipital: POz).

#### 3.4.2. The Relationship between EEG and Shooting Performance

In order to explore the relationship between EEG activity and shooting performance, multiple regression analysis was conducted with shooting score as the dependent variable and either theta or alpha or beta power (in different regions) under noncompetition as the predictor variable. The multiple regression analysis model was established as follows: $\hat{y}=b_{0}+b_{1}x_{1}+b_{2}x_{2}+b_{3}x_{3}+b_{4}x_{4}$, where, *x*_1_, *x*_2_, *x*_3_, and *x*_4_, respectively, represent the power of prefrontal, central, temporal, and occipital regions, and the output *y* represents the shooting score.

In statistical analysis, the multivariate solution was reported for the ANOVAs when assumption of sphericity was violated. The main effects test showed the results for the ANOVAs when assumption of sphericity was approved. In order to reflect the influence of each effect, Partial eta-squared (*η*_*p*_^2^) statistic was calculated as measures of effect size estimates. Significant effects were examined using post hoc Sidak tests.

## 4. Results

### 4.1. The Effect of Competition on EEG Power

#### 4.1.1. HR (Heart Rate)

HR, *t*(11)=1.933, *p*=0.079 > 0.05. Increased from noncompetition to competition, Table 1, but there was no significant difference.

#### 4.1.2. Shooting Accuracy

**Figure 3:**
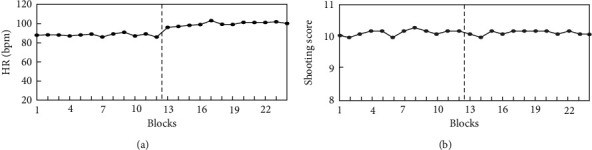
Time series for HR (a) and shooting score (b); noncompetition (1–12) and competition (13–24).

Shooting accuracy, *t*(11)=0.736, *p*=0.489 > 0.05. Shooting accuracy did not change obviously from the noncompetition to the competition state, Table 1, and the competition results were slightly lower than noncompetition.  Figure 3 shows the time series for shooting accuracy and HR.

#### 4.1.3. Theta Power

The 2 × 3 × 5(Condition × Epoch × Region) repeated measures MANOVA of theta data revealed a significant effect for Region, *F*(4,7)=13.164, *p*=0.002, *η*_*p*_^2^=0.883. Post hoc tests showed that theta power was higher over the prefrontal than over the frontal-central regions, and the theta power was higher over the temporal regions than over the frontal-central and occipital regions, Table 1. Finally, the interactions were revealed: *Con* *di* *tion* × *Epoch*, *F*(2,9)=1.1459, *p*=0.259, *η*_*p*_^2^=0.127; Condition × Region, *F*(4,7)=0.207, *p*=0.745, *η*_*p*_^2^=0.02; and Epoch × Region, *F*(8,3)=2.160, *p*=0.086, *η*_*p*_^2^=0.178. Compared with noncompetition, post hoc tests confirmed that theta power of the competition increased in the last second, whereas it did not change in other periods of time. The theta power increased from noncompetition to competition for occipital regions within three seconds before trigger pull, Table 1.

#### 4.1.4. Alpha Power

The 2 × 3 × 4(Condition × Epoch × Region) repeated measures MANOVA of alpha data revealed a main effect for Condition whereby alpha power increased from the noncompetition to the competitive condition, Table 1, *F*(1,10)=6.882, *p*=0.025, *η*_*p*_^2^=0.408. A major effect was also found for Region, *F*(3,8)=28.407, *p* ≤ 0.001, *η*_*p*_^2^=0.740. Post hoc analysis indicated that the alpha power was higher over the left temporal and occipital regions than over the frontal-central regions, Table 1. Finally, the interactions were revealed: Condition × Epoch, *F*(2,9)=0.334, *p*=0.671, *η*_*p*_^2^=0.032; Condition × Region, *F*(3,8)=0.732, *p*=0.561, *η*_*p*_^2^=0.215; Epoch × Region, *F*(6,5)=0.815, *p*=0.503, *η*_*p*_^2^=0.075; and Condition × Epoch × Region, *F*(6,5)=1.183, *p*=0.332, *η*_*p*_^2^=0.106. Post hoc tests confirmed that alpha power increased from noncompetition to competition for the frontal-central and left occipital regions, whereas it did not change in other regions. In addition, at −3000 to −2000 ms and −1000 to 0 ms, alpha power increased from noncompetition to competition for the frontal-central and left occipital regions, and at −2000 to −1000 ms, the alpha power increased from noncompetition to competition for the frontal-central regions.

#### 4.1.5. Beta Power

**Figure 4:**
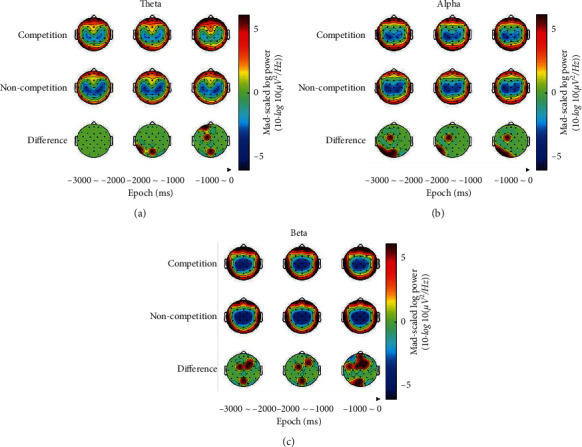
Scalp maps representing theta (a), alpha (b), and beta (c) power during Condition (noncompetition, competition) and Epoch (−3000 to −2000 ms, −2000 to −1000 ms, −1000 to 0 ms) averaged across subjects.

The 2 × 3 × 4(Condition × Epoch × Region) repeated measures MANOVA of beta data revealed a main effect for Condition whereby beta power increased from the noncompetition to the competitive condition, Table 1, *F*(1,10)=12.720, *p*=0.005, *η*_*p*_^2^=0.560. A major effect was also found for Region, *F*(3,8)=27.301, *p* ≤ 0.001, *η*_*p*_^2^=0.911. Post hoc tests showed that beta power was higher over the prefrontal region than over the mid-frontal, frontal-central, and mid-occipital regions, Table 1. Finally, the interactions were revealed: Condition × Epoch, *F*(2,9)=3.496, *p*=0.075, *η*_*p*_^2^=0.261; Condition × Region, *F*(3,8)=0.423, *p*=0.742, *η*_*p*_^2^=0.137; Epoch × Region, *F*(6,5)=1.107, *p*=0.367, *η*_*p*_^2^=0.100; and Condition × Epoch × Region, *F*(6,5)=0.471, *p*=0.657, *η*_*p*_^2^=0.052. Post hoc tests confirmed that beta power increased from noncompetition to competition for the mid-frontal, frontal-central, and mid-occipital regions, whereas it did not change in other regions, Table 1. In addition, at −3000 to −2000 ms and −2000 to −1000 ms, beta power increased from noncompetition to competition for the frontal-central and mid-occipital regions. Compared with noncompetition, the beta power of the competitive frontal and mid-occipital regions increased in the last second. Scalp maps for theta, alpha, and beta power were showed in Figure 4.

### 4.2. The Relationship between EEG and Shooting Performance

**Figure 5:**
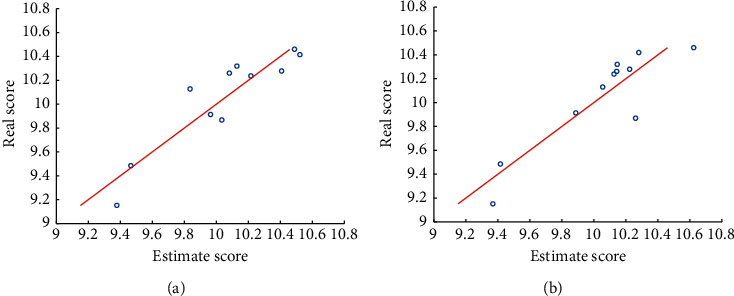
Predicted and true scores in shooting.

Multiple linear regression was conducted with shooting score as the dependent variable. The regression coefficient revealed a significant linear correlation of theta power (*R*^2^=0.844, *p*=0.014 ) and beta power (*R*^2^=0.812, *p*=0.022 ) on shooting score, but not for alpha power, Figure 5. The plot of the estimated scores versus the real scores for shooting was given in Figure 5, and the estimated scores were the linear combination of the power represented by *x*_1_, *x*_2_, *x*_3_ , and *x*_4_. These findings showed that the shooting performance was related to the distribution of brain power for prefrontal, central, temporal, and occipital regions.

### 4.3. Control Analyses

**Figure 6:**
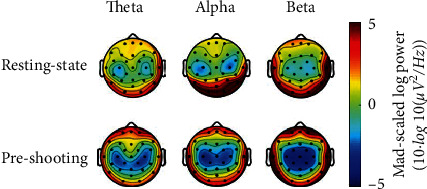
Scalp maps representing theta, alpha, and beta power averaged across subjects in resting-state (eyes open rest) and in preparation for shooting.

The eyes-open rest recordings conducted before the shooting task were used as nonshooting cortical activity (control condition). Theta, alpha, and beta power of resting-state (eye opening rest) were calculated and compared with the EEG activity of 3 seconds before shooting (power was averaged across competition and noncompetition). Theta power, alpha power, and beta power were subjected to repeated-measures MANOVA with the factors Condition and Region. The 2 × 4(Condition × Region) repeated measures ANOVA of theta data revealed a main effect for Region, *F*(3,8)=26.578, *p* ≤ 0.001, *η*_*p*_^2^=0.727 and Condition × Region, *F*(3,8)=5.407, *p*=0.011, *η*_*p*_^2^=0.357. The 2 × 4(Condition × Region) repeated measures ANOVA of alpha data revealed a main effect for Region, *F*(3,8)=41.919, *p* ≤ 0.001, *η*_*p*_^2^=0.807 and Condition × Region, *F*(3,8)=5.198, *p*=0.028, *η*_*p*_^2^=0.661. The 2 × 4(Condition × Region) repeated measures ANOVA of alpha data revealed a main effect for Region, *F*(3,8)=43.317, *p* ≤ 0.001, *η*_*p*_^2^=0.812 and Condition × Region, *F*(3,8)=15.985, *p* ≤ 0.001, *η*_*p*_^2^=0.615. Post hoc Sidak tests confirmed that theta, alpha, and beta topography for pre-shooting were different than that during nonshooting, Figure 6. During pre-shooting, power was higher over the prefrontal, temporal, and occipital regions than over the central regions, whereas, for nonshooting, power was higher over the temporal and occipital regions than over the central regions.

## 5. Discussion

This study examined pre-shooting EEG activities in a group of professional air rifle shooters at noncompetition and competition conditions. We collected and analyzed shooting accuracy data and EEG signals of 11 air rifle shooters and found that the HR and shooting accuracy did not change significantly between the competition load, suggesting that some compensatory strategies were adopted to cope with the increased demands of shooting under the competitive state [16, 33]. The analysis of theta power, alpha power, and beta power within the last three seconds before trigger pull revealed the effects of these strategies. Consistent with Pereira's report, our study did not find consistent differences in EEG power for competition in the groups [27], which could be because the subjects in this work were professional shooters, and the two groups had similar skill levels and could have the same neurocognitive strategies. However, we found the differences between the competitive and noncompetitive states.

Compared with the noncompetition, the theta power of the competition increased in the last second, whereas the occipital theta power was higher than the noncompetitive state within three seconds before trigger pull. These findings indicate that when shooting in a competitive environment, the resources were allocated more to the monitoring process in the last second before shooting, and the cognitive load increased, which is relevant for working memory. The closer to the moment of trigger pull, the more the shooters need to maintain a good mental state and focus the attention to the moment of the shot. This interpretation is compatible with reports that expert shooters can focus their attention correctly to the moment of the shot [6, 7], and a professional shooter may attain good performance when consciously turning his attentional focus to a core component of action [8].

Traditionally, alpha band power was considered to be inversely proportional to cortical activity. Compared with theta power, the EEG activity of alpha power increased from the noncompetitive state to the competitive state. This increase was mainly reflected for the frontal-central and left occipital regions, whereas it did not change in the other regions. This regional specificity can be interpreted as a strategy to inhibit cognitive process that was not relevant for shooting, particularly when shots were fired in a competitive environment. These findings suggest that the cognitive processes in the frontal-central and left occipital regions were related to shooting performance, which is compatible with previous research reports [16, 24, 34]. In the study of Loze et al., it was found that occipital EEG alpha power increased before best shots but decreased before worst shots. The report was similar to the findings of this article, which indicates that the increase in power of the left occipital regions can be interpreted to inhibit attention process that was not relevant for task specificity before shooting, thus reducing the attention to external visual stimulation [31, 35].

Since the alpha band signal is relevant for the whole brain excitation and professional information processing, the previous reports mainly study the EEG activity in the alpha band [36, 37]. Consider that beta-band brain rhythms are relevant for emotion and excitement [13, 18, 38]. EEG characteristics of beta band were analyzed in this work. Compared with theta and alpha bands, we found that the beta power was higher over the pre frontal, central, temporal, and occipital regions than over the central regions during the aiming period. The EEG activity of beta power increased from the noncompetitive state to the competitive state. This increase was mainly reflected for the mid-frontal, frontal-central, and mid-occipital regions, whereas it did not change in the anterior frontal regions. This may be because the evaluation of performance in the competitive environment induces certain emotions or cognitive activities of the subjects, which enable the EEG activities of beta band to cope with the demands of shooting. It is just a guess, and confirmation through further experimental validation is needed.

Whether the shooters are in the competitive or the noncompetitive state, the power is higher over the pre frontal, temporal, and occipital regions compared to other regions (central region) Figure 4. This result shows a regional specificity in which resources were allocated to each brain region in a timely way. This power distribution is specific for preparation for shooting, which is supported by control analyses (Figure 6). The EEG characteristics of the resting-state may be specific to shooting performance because subjects trained professionally may exhibit changes in the resting-state neurophysiological characteristics closely related to performance [12].

Although we have a certain understanding of the EEG activity of theta, alpha, and beta bands in the competitive environment, there are still some limitations in this work. On the one hand, the standing shooting posture of 10-meter air rifle was designed for this experiment, so it is impossible to infer whether the same results exist for other types of shooters or other shooting positions. On the other hand, the subjects selected in this experiment are professional shooters with a high technical level, so the conclusions of this study may not be applicable to novices or general shooters. Therefore, we recommend that future research should set a larger sample size and design more realistic competitive environment as experimental conditions to analyze the neurophysiological activities that are relevant for competition by more accurate research results.

## 6. Conclusions

In this work, we measured the EEG signals of 11 professional shooters and analyzed the correlation between EEG power and shooting performance. Results showed a significant linear correlation between shooting performance and prefrontal, central, temporal, and occipital regions in the beta band. In addition, we found that theta power in occipital region, alpha power in posterior frontal and left occipital region, and beta power in frontal and middle occipital regions were higher than those in the noncompetitive state. Our findings suggest that competition increases brain activity and changes the activation of individual brain regions. This strategy could guide shooters in aiming to automatically adjust these EEG characteristics by using neural feedback, which establishes a research foundation for the follow-up study of neural regulation to improve athletic grades. These two findings can be used to realize the basic prediction of shooting performance by monitoring EEG activity as well as providing further understanding of the neural mechanisms of shooting.

## Figures and Tables

**Table 1:** HR, shooting accuracy, alpha power, theta power, and beta power as a function of condition (noncompetition, competition).

	Non-competition M (SD)	Competition M (SD)	Δ (Competition-noncompetition)
HR (bpm)	85.164 (12.41)	94.928 (14.19)	9.764
Shooting accuracy (0.0–10.9)	10.052 (0.09)	10.048 (0.05)	−0.004
Theta power	0.241 (1.92)	1.415 (1.67)	1.174
Anterior frontal	3.223 (1.69)	4.504 (3.10)	1.281
Frontal-central	−0.135 (1.26)	1.074 (1.51)	1.209
Left temporal	2.256 (1.38)	3.757 (3.01)	1.501
Right temporal	2.005 (1.22)	2.783 (1.50)	0.778
Occipital	0.815 (1.26)	2.151 (1.27)	1.336
Alpha power	0.235 (0.77)	0.812 (1.00)	0.577^*∗*^
Frontal-central	−1.918 (1.34)	−1.081 (1.53)	0.837^*∗*^
Left temporal	3.394 (1.78)	4.161 (1.74)	0.767
Left occipital	2.631 (2.07)	3.637 (2.27)	1.006^*∗*^
Right occipital	2.827 (2.43)	3.390 (1.89)	0.563
Beta power	0.265 (0.614)	1.100 (0.71)	0.835^*∗∗*^
Anterior frontal	2.951 (3.08)	5.336 (3.22)	2.385
Mid-frontal	−0.544 (1.38)	0.488 (1.63)	1.032^*∗*^
Frontal-central	−3.303 (1.01)	−2.116 (1.35)	1.187^*∗*^
Mid-occipital	−0.759 (1.71)	0.342 (1.23)	1.101^*∗*^

*∗*=*p* < 0.05, *∗∗*=*p* < 0.01, Unit : 10*·* log 10(*μ*V^2^/Hz).

## Data Availability

The data used to support the findings of this study are restricted by the ethics committee of Tsinghua University in order to protect subject privacy. The data are available from the corresponding author for researchers who meet the criteria for access to confidential data.
